# HPV-Mediated Radiosensitivity in Oropharyngeal Squamous Cell Carcinoma: Molecular Mechanisms and Cellular Pathways

**DOI:** 10.1007/s11912-025-01666-2

**Published:** 2025-04-11

**Authors:** Allen M. Chen

**Affiliations:** Department of Radiation Oncology, Irvine, Chao Family Comprehensive Cancer Center, University of California, 101 The City Drive, Building 23, Orange, CA 92868 USA

**Keywords:** Human papillomavirus, Oropharynx cancer, Molecular, Radiation, Sensitivity

## Abstract

**Purpose of Review:**

While the oncogenic potential of HPV has been well-established in other disease sites (e.g. cervix, vulva, anus), it is increasingly evident that a significant proportion of oropharyngeal cancer cases are related to the virus. Although considerable progress has been made in the understanding of this disease with respect to its underlying biology and clinical behavior, numerous questions persist. From a therapeutic standpoint, HPV-positive oropharyngeal cancer has been shown to be more radiosensitive than HPV-negative disease. However, how HPV mediates this radiosensitivity is relatively uncertain.

**Recent Findings:**

Given that it has been firmly established that patients with HPV-positive oropharyngeal cancer have a significantly improved prognosis as a result of their exquisite response to radiation and can be treated with less-than-standard doses, logical questions pertain to how HPV confers this benefit to infected patients. Although the exact reason for the improved radiosensitivity of HPV-positive oropharyngeal carcinoma is unclear, multiple theories have been proposed. Indeed, it is likely that no single explanation exists for the increased radiosensitivity, and instead, HPV likely exerts its influence through a cascade of activated pathways at both the cellular level and tumor microenvironment. As will be discussed in this review, the proposed mechanisms for HPV-induced radiation response have generally centered on the disruption of such cellular pathways as DNA repair, cell cycle checkpoints, metabolic-induced stress, immunology, and cancer stem cells.

**Summary:**

Given that HPV-positive oropharyngeal cancer is increasingly recognized as a public health problem, the search to better understand its unique biological radiosensitivity has important societal and treatment-related implications.

## Introduction

The worldwide incidence of human papillomavirus (HPV)-positive oropharyngeal squamous cell carcinoma has increased dramatically in recent years reaching epidemic-like proportions in developed countries [1]. For many patients, radiation therapy, either delivered as primary treatment or postoperatively, is recommended. Given the data that has accumulated demonstrating that patients with HPV-positive oropharyngeal cancer have a favorable prognosis compared to their HPV-negative counterparts, interest in understanding the biological basis for these differences has grown. Moreover, with the recognition that HPV-positive oropharyngeal carcinoma represents a remarkably radiosensitive subtype of head and neck cancer, studies evaluating potential molecular mechanisms underlying this radiosensitivity has intensified [2]. From a clinical standpoint, studies investigating whether treatment can be differentiated based on the presence or absence of HPV are based on a plethora of biological data showing that HPV-positive oropharyngeal cancer is exquisitely sensitive to treatment [3]. Indeed, correlative biomarker studies have so convincingly confirmed HPV status as the single most important predictor of radiation response among oropharyngeal cancer patients that separate staging systems have been developed and validated for HPV-positive and HPV-negative oropharyngeal cancer by the American Joint Committee on Cancer (AJCC) [4, 5]. Furthermore, findings from prospective trials have demonstrated that patients with HPV-positive oropharyngeal cancer can be treated with reduced doses of radiation due to the unique biological characteristics of the tumor [6–8]. The rationale for de-escalation is based on the recognition that HPV-positive oropharyngeal cancer responds favorably to radiation and hence patients can possibly be spared the toxicity of high-dose radiation. However, the biological reasons underlying the enhanced response of these tumors to radiation remain somewhat elusive. Although it is generally recognized that HPV-positive oropharyngeal cancer arises in the setting of non-smoking, this observation in itself does not explain the radiosensitivity of these tumors per se. Instead, investigators are focused on understanding the underlying biological mechanisms driving treatment response so that they can be exploited in the clinical setting. Given the tremendous interest in de-escalation strategies around the world, the molecular basis for such strategies constitutes the focus of this review.

## The Evidence

### HPV Biology

From a microscopic standpoint, HPV is a relatively small epitheliotropic DNA virus that is involved in the etiology of several human malignancies including those of the oropharynx. Indeed, an increasing proportion of all oropharyngeal carcinomas are associated with the high-risk HPV subtype, most commonly HPV-16 and HPV-18. Unlike in cervical cancer where HPV-DNA is generally integrated into the host cell genome, HPV-positive oropharyngeal cancer is notable for episomal expression. Through the activation of the oncoproteins E6 and E7, the HPV-DNA promotes genetic instability by inactivating the tumor suppressor and cell cycle checkpoint proteins p53 and pRb, respectively, thus leading to carcinogenesis. Although the E6 and E7 oncoproteins seem to be the logical sources for mediating radiosensitivity, studies analyzing the roles of these oncoproteins with respect to radiation response have suggested that other pathways are more likely involved. Consequently, there is no conclusive evidence demonstrating a firm role of these oncoproteins in mediating radiation response. For example, DeWeese et al. showed that while low-dose irradiation to human colon carcinoma cells engineered to express E6 and E7 showed increased levels of p53 and enhanced cell cycle arrest at G1 and G2, no difference in clonogenic survival was ultimately observed [9]. Another study using cervical carcinoma cell lines showed no change with respect to intrinsic radiosensitivity when E6 and E7 were knocked down [10]. Hampson et al. similarly showed that HPV-negative cervical carcinoma cell line shifted, paradoxically, to a more radioresistant phenotype when HPV16 E6 was overexpressed [11]. These historic studies, in aggregate, have suggested that radiosensitivity involves much more than just the viral oncoproteins expressed through HPV infection and that a more complex network of pathways is likely responsible for mediating radiation response.

### Relationship between HPV and p16

Although HPV-positive oropharyngeal cancer is now recognized to be a distinct entity, it is important to recognize that the AJCC staging system, similar to most clinical trials, have considered p16-positivity to be equivalent to HPV-positivity. As a result, practical questions of how to define p16-positivity with respect to staining pattern and/or intensity complicate this issue. Relatedly, studies have now shown that the discordance between p16 and HPV may be significant in oropharyngeal cancer. This is relevant because it is now established that patients with p16–negative/HPV-positive or p16-positive/HPV–negative disease display a significantly worse prognosis than patients with p16-positive/HPV-positive oropharyngeal cancer, and a significantly better prognosis than patients with p16–negative/HPV-negative oropharyngeal cancer [12]. These observations have implications not only with respect to treatment but may also provide insight into the underlying biology of the tumor. For instance, studies from across a variety of difference cancers have shown that the p16 gene and/or protein is associated with radiosensitivity. More notably, Molkentine et al. showed that p16 converts cells to a more radiosensitive phenotype and this mechanism is mediated through a ubiquitin-dependent signaling pathway, linking high levels of this protein to increased activity of the transcription factor SP1, increased HUWE1 transcription, and degradation of ubiquitin-specific protease 7 and TRIP12 [13]. Through a series of elegant experiments, the investigators showed that activation of this pathway in head and neck cancer cell lines led to decreased homologous recombination and improved response to radiation. This p16-driven axis provided evidence of a directly link between p16 to DNA damage repair and radiosensitivity through a targetable ubiquitin-mediated degradation pathway.

### Mechanisms of Radiosensitivity

While the exact mechanism of HPV-mediated radioresponse is thus currently unclear, numerous models have been proposed in the setting of oropharyngeal cancer. It is likely that no single explanation exists for the increased radiosensitivity; instead, HPV likely exerts its influence through a multitude of activated pathways at both the cellular level and tumor microenvironment. It is further likely that these pathways are overlapping and interact with one another in ways that are complex. While scientists have long known that HPV infection of the host tumor cell leads to the degradation of the p53 and pRb proteins by the viral products E6 and E7, how this action triggers a cascade of molecular events which renders the host tumor cell more susceptible to radiation-induced apoptosis has remained elusive [14]. As will be discussed, the proposed mechanisms for HPV-induced radiation response have generally centered on the disruption of such cellular pathways as DNA repair, cell cycle checkpoints, metabolic-induced stress, and cancer stem cells.

The data on the interaction between HPV and DNA repair in mediating radiation sensitivity continues to prove provocative. Several studies have shown that the capacity of DNA repair might be hindered by HPV as measured by the persistence of double-strand breaks [15–17]. Using immunofluorescence staining with anti-γH2AX and 53BP1 antibodies, it has been shown that residual γH2AX/53BP1 lesions after irradiation were more pronounced in HPV-positive cancer cells indicating alteration of homologous recombination and nonhomologous end-joining [18, 19]. Zhang et al. showed that the levels of γ-H2AX foci formation and retention were time and cell line-dependent—with distinct differences between HPV-positive and HPV-negative head and neck squamous cell carcinoma cell lines [15]. More specifically, HPV-positive cell lines showed prolonged γ-H2AX foci formation up to 48 h after radiation thereby suggesting that these cells were less able to repair the radiation induced double-strand breaks as a result of deficiencies in DNA repair and hence more likely to undergo mitotic cell death during the cell cycle. Further evidence supporting a role of DNA repair is based on observations that the expression of SMG-1, a key protein involved in DNA damage response, is negatively correlated with HPV-positive oropharyngeal tumors [20]. In vitro, decreased SMG-1 expression was seen in cell lines transfected with E6/E7 and such cells had enhanced radiosensitivity. Lastly, the potential of p16 to promote radiosensitivity has also been explored. Dok et al. showed that p16 overexpression leads to inhibition of Rad51, an important protein integral to homologous recombination, which makes DNA repair subjected to errors [21]. While it is increasingly evident that HPV interacts with a number of different host DNA repair pathways that potentially render a tumor cell more radiosensitive, the specific mechanisms still remain under active investigation.

The effects of HPV on the cell cycle of the host tumor cell have also been described and thought to possibly play a role in mediating radiation response. Most pointedly, it has been suggested by numerous investigators that HPV is associated with prolonged G2/M arrest, thus increasing the proportion of tumor cells in the most radiosensitive part of the cell cycle [22]. For instance, Pang et al. showed that the E6 oncoprotein may trigger G2/M arrest, thus leading to a proclivity in diverting tumor cells to programmed cell death [23]. Similarly, Kimple et al. demonstrated that HPV-positive cell lines derived from squamous cell head and neck cancer exhibited greater intrinsic radiosensitivity characterized by prolonged G2-M cell-cycle arrest and increased apoptosis compared to HPV-negative cell lines [24].

Several groups have also suggested that HPV may decrease cellular hypoxia resulting in enhanced radiosensitivity. For instance, Knuth et al. showed HPV-positive head and neck squamous cell carcinoma had high levels of activation of the HIF pathway and adaptation to HIF-1α upregulation, pathways known to affect hypoxia [25]. Notably, in the Danish Head and Neck Cancer Group (DAHANCA) 5 trial, HPV-negative cells were associated with a higher prevalence of hypoxia-related modifications than HPV-positive cells, based on the expression level of hypoxia markers [26, 27]. The investigators thus concluded that low levels of hypoxia in HPV-positive cells may have contributed to their enhanced radiosensitivity [28]. In addition, this evidently explained the lack of benefit observed in HPV-positive oropharyngeal cancer patients treated with nimorazole, a hypoxia modifier.

Clinical-translational studies evaluating the potential role of tumor metabolism as a marker of treatment response have also yielded valuable insight on this disease. For instance, the use of mid-treatment functioning imaging of metabolic activity has been proposed as a means of assessing radiation response for HPV-positive oropharyngeal cancer [29]. Investigators from Duke University conducted a prospective study in which 62 patients treated by chemoradiation underwent ^18^F-fluorodeoxyglucose positron emission tomography (PET)/computed tomography (CT) scans at simulation and after 2 weeks at a radiation dose of approximately 20 Gy [30]. On recursive portioning analysis, several PET-related variables obtained at 2-weeks mid-treatment including SUV-max and SUV-40% were shown to be prognostically significant—validating the premise that metabolic information could be used to identify patients with favorable biology (i.e. more radiosensitive tumors) early during treatment. Investigators from the University of Michigan similarly presented the results of a phase II study in which the findings from mid-treatment PET/CT was used to stratify patients with HPV-positive oropharyngeal cancer to 54 Gy or 70 Gy with concurrent chemotherapy [31]. More specifically, if the metabolic tumor volume showed a greater than 50% reduction, patients proceeded to the de-escalated dose of 54 Gy. With a median follow-up of 32 months for the patients receiving 54 Gy, the 2-year local–regional control was shown to be 90%, suggesting that mid-treatment PET is a reliable surrogate of radiosensitivity.

Other investigators have shown that hypoxia monitoring using novel radiotracers can be useful for identifying patients with more radiosensitive tumors in the setting of HPV-positive oropharyngeal cancer. In a prospective study utilizing fluoromisonidazole-positron emission tomography (F-MISO-PET) to image hypoxia during radiation, researchers from Memorial Sloan Kettering Cancer Center showed that radical reduction in radiation dose to 30 Gy for those with no pretreatment hypoxia or in whom hypoxia had resolved within the first 2 weeks of initiating radiation might be feasible [32]. In their prospective phase II study, 158 patients first underwent surgical removal of disease at their primary site, but not of gross disease in the neck [33]. A baseline ^18^F-fluoromisonidazole positron emission tomography scan was used to measure tumor hypoxia and was repeated 1–2 weeks mid-treatment. Patients with nonhypoxic tumors received 30 Gy (3 weeks) with chemotherapy, whereas those with hypoxic tumors received standard chemoradiotherapy to 70 Gy (7 weeks). With a median follow-up time of 38 months, the 2-year PFS was 94% and overall survival was 100%, respectively for the 30 Gy cohort suggesting that this technique can successfully identify tumors that are more radiosensitive and that dramatic reductions in dose can be feasibly used with the incorporation of hypoxia-based targeted treatment.

Given that p53 plays a prominent role in regulating metabolic pathways and also acts as a facilitator of DNA repair by halting the cell cycle to allow time for the repair machineries to restore genome stability, interest has emerged in its potential role in mediating radiation sensitivity. Data has emerged that this mutations in this tumor suppressor gene is linked to a high mutational tumor burden and increased rates of recurrence in head and neck cancer [34–36]. Tinhofer et al. showed through next generation sequencing that p53 mutations could identify a high-risk group of patients with poor outcomes after chemoradiation [37]. Another study from Japan showed that patients with p53 mutated/HPV-positive tumors had a significantly worse outcome than those with p53 wild-type/HPV-positive oropharyngeal cancer [38].

The role of cancer stem cells in promoting radiation resistance for HPV-positive oropharyngeal cancer has also been recognized. Indeed, the presence of a subpopulation of cells within the tumor that has the ability to self-renew, differentiate, and generate tissue to propagate the tumor is classically believed to be responsible for recurrence and resistance to treatment. In a series of experiments, Vlashi et al. showed that that the improved radiosensitivity of HPV-positive head and neck cancer might be due to the lower frequency of cancer stem cells as measured by low proteasome activity. In addition, the investigators showed that HPV-positive cell lines exhibited a marked decreased capacity to engage in radiation-induced dedifferentiation compared to HPV-negative head and neck cancer [39]. Similarly, researchers from the Netherlands showed that stem cell expression as measured by CD44 and CD98 was significantly lower in HPV-positive oropharyngeal squamous cancer cells than in HPV-negative cells—and proposed that differential activity of cancer stem cells could be responsible for the divergence in radiosensitivity [40].

There is tremendous interest in evaluating the immunological response of radiation in the setting of HPV-positive oropharyngeal cancer. Studies have suggested that radiation therapy enhances the host immune response to viral antigens which are expressed on the cancer [41–43]. Numerous studies have confirmed an immunologic mechanism to HPV-mediated radioresponse by demonstrating that the extent of tumor-infiltrating lymphocytes is associated with clinical outcome among patients treated for HPV-positive oropharynx cancer [44, 45]. Others have shown that the levels of leukocytes in the peripheral blood are of importance in mediating response to radiation [46, 47]. More recently, the density and pattern of immune infiltrates in the tumor microenvironment has been proposed to be a byproduct of the HPV activation process in oncogenesis. Relatedly, the presence of regulatory T cells and PD-1( +) T cells and the levels of PD-1( +) cells were positively correlated with a favorable clinical outcome in HPV-positive compared to HPV-negative head and neck cancers [48–50]. While speculative, this may reflect prior immune response in HPV-positive tumors, and radiation may possess a role in helping to re-activate this immune response. The potential role of tumor-associated macrophages and regulatory T cells in mediating HPV-related radioresponse is also increasingly being investigated [51–53]. These studies have demonstrated the importance of the microenvironment and its interaction with tumor cells in mediating radiation response in the setting of HPV-positive oropharyngeal carcinoma. In a series of elegant experiments, Spanos et al. demonstrated the importance of an intact immune system in mediating radiation response in the setting of HPV-positive head and neck caner [41]. Using an in vivo mouse model, the researchers first showed that HPV-positive tumors were more sensitive to radiation and exhibited complete clearance at 20 Gy, compared to HPV-negative counterparts, which showed persistent growth. However, the effects of radiation were mitigated considerably using immunodeficient mice. Furthermore, adoptive transfer of wild-type immune cells into immunodeficient mice restored HPV-positive tumor clearance with treatment.

Given that HPV-positive oropharyngeal caner has been increasingly well-established to be an immunologically “hot” tumor, the identification of other key immune mediators in the microenvironment may yield additional insight on the mechanisms of radiosensitivity. For instance, the incorporation of other immunologic biomarkers such as PD-1/PDL-1 in conjunction with HPV has also been studied as a more powerful means to refine risk stratification [54–56]. Corredor et al. recently employed image processing and machine learning to develop an imaging biomarker that quantitatively characterized the spatial patterns of tumor-infiltrating lymphocytes and surrounding nucleated cells in digitized hematoxylin and eosin slides of HPV-positive oropharyngeal cancer patients [54]. The investigators then showed how this model could be implemented in current staging systems to refine prognostication and to aid in the identification of patients who might be more radiosensitive than others.

In the largest study attempting to define immune-based signatures for HPV-positive oropharyngeal cancer, Zeng et al. utilized clinical data from 906 patients to develop an innovative scoring system (UWO3) based on tumor immunology [57]. By considering the immune population of the tumor microenvironment including such elements as T-lymphocytes, B-lymphocytes, natural killer cells, myeloid dendritic cells, neutrophils, and endothelial cells in conjunction with the expression of specific immunological genes, the investigators developed the UWO3 scoring system which they proposed could stratify patients into 3 distinct immune classes (immune rich, mixed, and immune desert). Using a test set of 5 independent cohorts of 863 patients, the researchers validated the prognostic significance of their classification system, thereby suggesting that the contributions of various immunologic factors play a significant role in mediating radiation response. In another study, investigators from Germany demonstrated the potential importance of human leukocyte antigen (HLA) in mediating differential responses to treatment in the setting of HPV-positive oropharyngeal cancer [58]. Using DNA samples from peripheral blood specimens from 94 patients with HPV-positive disease treated by a variety of different methods, the researchers conducted HLA typing with sequence-specific oligonucleotides. On statistical analysis, the distribution of certain HLA traits could be correlated with progression-free survival and overall survival. Notably, approximately three-quarters of the population received radiation, suggesting that an immunologic basis could explain the differences in observed outcome.

More recently, the transcription factor, nuclear factor-kappa B (NF-*κ*B), has been demonstrated to be involved in the mechanisms of HPV-mediated sensitivity to radiation [59, 60]. Given that NF-κB regulates multiple aspects of innate and adaptive immune functions and is well-known as a pivotal mediator of the immunological response to radiation, investigators have proposed that this pathway might be of utility for the development of prognostic models. Shrank et al. showed that HPV-positive oropharyngeal cancer cells with constitutive activity of NF- κB have a distinct pattern of gene expression and markedly unique mutational and methylation profiles [61]. Moreover, the investigators also demonstrated that NF- κB was the only gene set among 11,843 analyzed that successfully segregated patients into prognostic groups associated with differences in disease-free survival. On multivariate analysis, NF- κB status was not only prognostic while controlling for stage and smoking history but emerged as the most powerful predictor of disease-free survival, suggesting that this pathway might be involved in mediating radiation sensitivity in HPV-positive oropharyngeal cancer.

The clinical implications of understanding the molecular basis for radiosensitivity in the setting of HPV-positive oropharyngeal cancer are immense. One especially important issue relates to the increasing interest in using de-escalated radiation doses for the treatment of HPV-positive oropharyngeal cancer and the resultant need to identify patients with radioresistant tumors that might not be appropriate candidates for this strategy. Conversely, the ability to identify patients with radiosensitive tumors that can safely proceed to de-escalation is equally as important as the reassurance provided is valuable for both providers and patients. Ultimately, advances in individualized approaches to de-escalation will be defined by improvements in understanding the biological basis for recommending de-escalated radiation. It is likely that further advances in de-escalation will involve risk-adapted approaches which will incorporate a combination of clinical, radiological, and biological data—helping to apply principles of precision medicine to state-of-the-art treatment.

While the vast majority of studies to date have focused on the molecular mechanisms covered herein, including those related to DNA repair, cell cycle arrest, metabolism, hypoxia, cancer stem cells, and immunology, it is likely that additional pathways will be discovered that also play a contributory role in mediating the radiosensitivity of HPV-positive oropharyngeal cancer. Given that it was only relatively recently that HPV was shown to be an etiologic agent in the pathogenesis of oropharyngeal cancer, further work will yield additional insight into helping scientists and clinicians better understand how the virus contributes to radiosensitivity.

## Conclusion

**Fig. 1:**
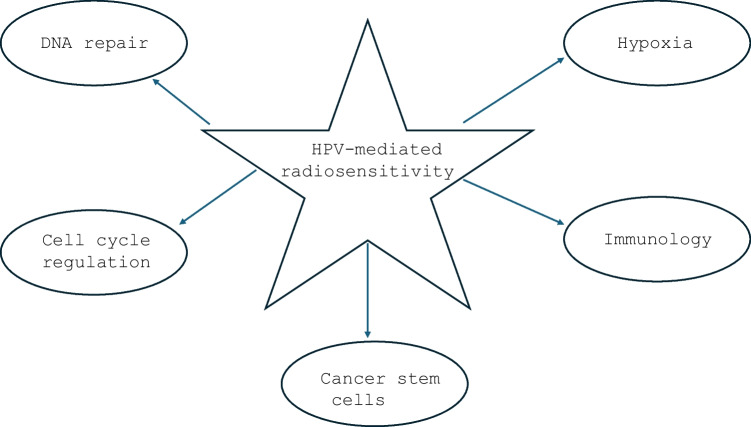
Core themes pertaining to the mediation of radiation response for HPV-positive oropharyngeal cancer

While the specific mechanism underlying the radiosensitivity of HPV-positive oropharyngeal cancer still remains somewhat elusive, considerable progress has been achieved in understanding its associated biological basis and has generally centered on the themes covered in this review (Fig. 1). The clinical repercussions are immense, as a better understanding of how HPV promote radiosensitivity will allow investigators to customize treatment for patients based on the inherent characteristics of their tumors. Ongoing scientific research combined with clinical trials evaluating de-escalated radiation have the potential to provide further insights into how the molecular biology of this disease can uniquely be exploited to help patients in the future.Unraveling the mechanisms responsible for the radiosensitivity of HPV-positive oropharyngeal cancer has the potential to lead to translational breakthroughs in clinical practice. Understanding why some tumors are more radiosensitive than others will allow for more meaningful risk stratification with respect to prognostication and also will permit the individualization of treatment based on one’s biological characteristics. Notably, numerous molecular pathways have been proposed to explain the radiosensitivity of HPV-positive oropharyngeal cancer, and investigation is warranted to better elucidate which mechanisms are of the most critical relevance. Additionally, prospective clinical studies incorporating the biological information from radiosensitivity data have the potential to validate these models further.

## Key References

- Mehanna H, Taberna M, von Buchwald C, et al. Prognostic implications of p16 and HPV discordance in oropharyngeal cancer (HNCIG-EPIC-OPC): a multicentre, multinational, individual patient data analysis. Lancet Oncol 24: 239–251.
  - An analysis of multicentre data on how HPV status may potentially interact with p16 in oropharyngeal cancer.
- Dok R, Kalev P, Limergen EJ, et al. p16INK4a impairs homologous recombination-mediated DNA repair in human papillomavirus-positive head and neck tumors. Cancer Res 2014; 74: 1739–1751.
  - Basic research study investigating potential mechanisms of p16-mediated radiosensitivity.
- Lee NY, Sherman EJ, Schoder H, et al. Hypoxia-directed treatment of human papillomavirus-related oropharyngeal carcinoma. J Clin Onol 2024; 42: 940–950.
  - Prospective study of hypoxia-target radiation for HPV-positive oropharyngeal cancer.
- Vlashi E, Chen AM, Boyrie S et al. Radiation-induced dedifferentiation of head and neck cancer cells into cancer stem cells depends on human papillomavirus status. Int J Radiat Oncol Biol Phys 2016; 94: 1198–1206.
  - Basic study demonstrating the relationship between HPV and cancer stem cells.
- Wang J, Sun H, Zeng Q, et al. HPV-positive status associated with inflamed immune microenvironment and improved response to anti-PD-1 therapy in head and neck squamous cell carcinoma. Sci Rep 2019; 13404.
  - Translational study showing that stratification of HPV-positive oropharyngeal cancer based on immune profiles may be prognostically significant.

## Data Availability

No datasets were generated or analysed during the current study.

## Declarations

### Transparency Statement

The lead author (AC) affirms that this manuscript is an honest, accurate, and transparent account of the study being reported; that no important aspects of the study have been omitted; and that any discrepancies from the study as planned (and, if relevant, registered) have been explained.
